# The Effects of Emotional Dysregulation and Impulsivity on Suicidality in Patients with Bipolar Disorder

**DOI:** 10.1192/j.eurpsy.2022.1049

**Published:** 2022-09-01

**Authors:** F. Kulacaoglu, F. Izci

**Affiliations:** 1 Istanbul Bakirkoy Prof Dr. Mazhar Osman Research and Training hospital for mental health and neurological diseases, Psychiatry, istanbul, Turkey; 2 Istanbul Erenkoy Training and Research Hospital for Psychiatry and Neurological Diseases, Psychiatry, Istanbul, Turkey

**Keywords:** bipolar disorder, emotional dysregulation, Impulsivity, Suicide

## Abstract

**Introduction:**

Bipolar disorder (BD) is a prevalent, often severe, and disabling illness that affects about 1-5% of the general population and has the highest risk of suicide among psychiatric disorders. Impulsivity and, emotion dysregulation have been emphasized due to their role in facilitating suicide acts among those with suicide ideation.

**Objectives:**

This study aimed to examine the relationship between emotional dysregulation, impulsivity, and suicidality in patients with bipolar disorder by comparing bipolar patients with healthy individuals

**Methods:**

The study included 85 patients (59 women, 26 men) with bipolar disorder and education and age-matched 65 (44 women, 21 men) healthy volunteers. The patient group was divided into 3 subgroups according to have suicide attempt history, have suicide ideation without attempt, and have neither suicide attempt nor ideation. Sociodemographic Form, The Difficulties in Emotion Regulation Scale (DERS), Barratt Impulsivity Scale (BIS-11), Scale for Suicide Ideation, Suicide Behaviors Questionnaire scales were applied to the participants.

**Results:**

Patients with bipolar disorder had significantly higher scores for emotion dysregulation and impulsivity than the healthy control group. A statistically significant correlation was found between emotion dysregulation, impulsivity, suicide ideation, and suicide behavior scores. DERS Total and Barratt Total scores were found higher for bipolar patients with suicide attempts than bipolar patients with suicide ideation and bipolar patients with neither attempt nor ideation. The hierarchical regression analysis has indicated that strategies, clarity, and non-planing impulsiveness were the predictors of suicide ideation in bipolar patients.

**Conclusions:**

The results suggested a strong association between emotional dysregulation, impulsivity with suicide ideation, and behavior in patients with bipolar disorder.

**Disclosure:**

No significant relationships.

